# Prevalence of NAFLD in Healthy and Young Male Individuals

**DOI:** 10.5402/2011/363546

**Published:** 2011-06-08

**Authors:** Asif Niaz, Zafar Ali, Shaista Nayyar, Naureen Fatima

**Affiliations:** PNS Shifa Hospital, Dha phase II, Karachi 74200, Pakistan

## Abstract

*Introduction*. Nonalcoholic fatty liver disease (NAFLD) is an important cause of liver disease in adults and the most common cause of liver disease in children (Lavine and Schwimmer 2004). The abnormalities include increased liver fat without inflammation (steatosis) and nonalcoholic steatohepatitis (NASH). NASH may lead to fibrosis, cirrhosis, and ultimately liver failure if it is not treated (Matteoni et al. 1999). The objective of the study is to estimate the magnitude of the problem which will help us to formulate strategies in managing the potentially difficult problem. *Materials and Methods*. We included 1000 individuals between the ages of 30 and 50 years who came for annual checkup. The patients with other comorbidities like diabetes, ischemic heart disease, chronic liver disease, or renal diseases were excluded from the study. History of alcohol ingestion was also taken; any individual with history of alcohol intake was also excluded. All of them underwent investigations including CBC, LFTs, height and weight. The individuals who were found to have increased ALT (50 to 150 u/L) further underwent investigations including ultrasound of abdomen hepatitis b and c serology RA and ANA antibodies. All the individuals who were found to have viral or autoimmune illness were excluded from the study. The individuals having raised ALT levels and ultrasound evidence of fatty liver were taken. *Results*. 13.5% of the individuals were found to have NAFLD among those selected for the study. *Conclusion*. Mass campaign regarding physical and dietary measures needs to be undertaken in general masses regarding the gravity and potential prevention of the disease.

## 1. Introduction

Nonalcoholic fatty liver disease (NAFLD) is a clinical spectrum of liver abnormalities associated with obesity, a common liver disease, and also the most common cause of liver disease in children [1]. The abnormalities include steatosis (increased liver fat without inflammation) and nonalcoholic steatohepatitis (NASH, increased liver fat with inflammation). NASH may lead to fibrosis, cirrhosis, and ultimately liver failure if it is not treated [2].

The pathogenesis of NAFLD in overweight and obese individuals is not exactly known. It appears to be related to insulin resistance. There are important clinical associations between NAFLD and elements of the metabolic syndrome, including insulin resistance, dyslipidemia, and hypertension, independent of the degree of obesity. Therefore, individuals with NAFLD should be carefully evaluated for each of these comorbidities and have counseling at national level about nutrition, physical activity, and tobacco use to help prevention of the development of cardiovascular disease and type 2 diabetes mellitus along with chronic liver disease. With the increased prevalence of childhood obesity, NAFLD is increasingly seen in children also. It is more common in males [3, 4]. NASH may progress to cirrhosis in up to 20 percent of patients [2, 5]. NASH is now recognized to be a leading cause of cryptogenic cirrhosis [5].

 The prevalence depends upon the population (i.e., referral community, ethnic group) and the definition (e.g., height of aminotransferase elevation and/or ultrasonographic findings). Imaging may confirm the presence of fatty liver, indicated by increased echogenicity. However, the severity of liver involvement does not correlate with radiographic features, clinical features, or the degree of elevation of liver transaminases. 

Asian population as a race is a risk factor for diabetes mellitus and obesity because increase in incidence of NASH in this ethnic group. This study was designed to find out the prevalence of NAFLD in our healthy population, and this will further lead us in formulating mass campaign regarding physical and dietary measures that need to be under taken in general masses regarding the gravity and potential prevention of the disease.

## 2. Materials and Methods

The study was conducted at PNS Shifa Hospital, Karachi which is a tertiary care hospital. We included 1000 consecutive individuals between the ages of 30 and 50 years who came for medical checkup in the last three months of 2010. The patients with other comorbidities like diabetes, ischemic heart disease, chronic liver disease and renal diseases were excluded from the study. History of alcohol ingestion was also taken; any individual with history of alcohol intake was also excluded. After fulfilling the inclusion and exclusion criteria, 952 subjects were selected. They undergo investigations including CBC, LFTs, height, and weight. The individuals who were found to have increased ALT (50 to 150 uL) further underwent investigations including ultrasound of abdomen hepatitis b and c serology RA and ANA antibodies. The individuals who were found to have viral or autoimmune illness were again excluded from the study. The individuals having raised ALT levels were taken as positive results. They then subjected to ultrasound of abdomen to find out the radiological evidence of fatty change. Liver biopsy was not done as most of the patients declined liver biopsy.

## 3. Results

**Figure 1:**
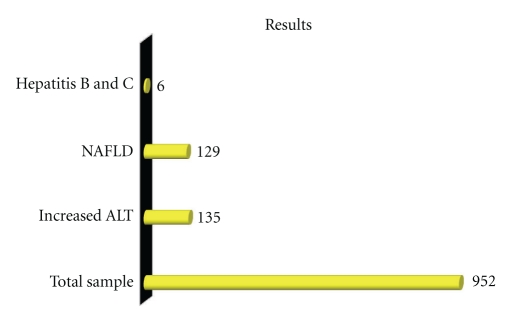
Prevalence of NAFLD in healthy and young male individuals.

**Figure 2:**
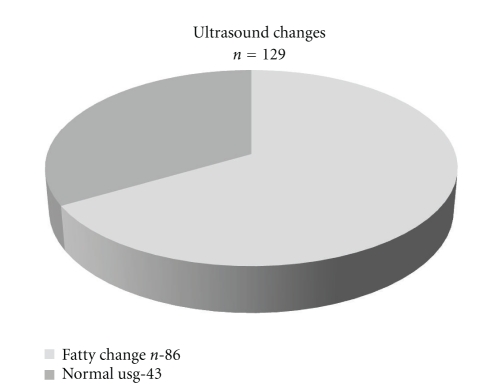
Ultrasound changes of NAFLD.

A total of 952 patients were selected after fulfilling inclusion and exclusion criteria. 135 patients had increased ALT levels. Six patients found to have either HBV or HCV serology positive. So a total of 129 patients had NAFLD (Figure 1). 113 patients have ALT between 50 and 100 U/L and 16 had ALT between 100 and 150 U/L. The average BMI of selected population for the study was 24.4, while average BMI was 27.1 in patients with increased ALT (Table 1). 86 patients having raised ALT also had fatty change on ultrasound examination (Figure 2). 

## 4. Discussion

 Nonalcoholic fatty liver disease is a spectrum of disorders that range from simple hepatic steatosis without significant inflammation or fibrosis to nonalcoholic steatohepatitis with varying degrees of inflammation and fibrosis. It is an clinical entity with histological features that resemble alcohol-induced liver injury, but by definition occurs in patients with no history of alcohol intake. The histological spectrum ranges from fat accumulation in hepatocytes without concomitant inflammation or fibrosis to hepatic steatosis associated with an inflammatory component that may be associated with fibrosis referred to as nonalcoholic steatohepatitis (NASH). This may progress to cirrhosis in up to 20 percent of patients. NASH is now recognized to be a leading cause of cryptogenic cirrhosis. Although the etiology of NASH is unknown, it is frequently associated with obesity, type 2 diabetes mellitus, and hyperlipidemia. The combination of abdominal obesity, hypertension, diabetes, and dyslipidemia has been called the metabolic syndrome [6, 7].

The pathogenesis of nonalcoholic fatty liver disease has not been fully elucidated. The most widely supported theory implicates insulin resistance [8] as the key mechanism leading to hepatic steatosis, and perhaps also to steatohepatitis. Another mechanism may be due to additional oxidative injury and is required to manifest the necroinflammatory component of steatohepatitis. Hepatic iron [9], leptin [10], antioxidant deficiencies [11, 12], and intestinal bacteria have all been suggested as potential oxidative stressors. The patients lack a history of significant alcohol consumption but have liver biopsy findings indistinguishable from alcoholic steatohepatitis.

NASH is considered to be a subset of nonalcoholic fatty liver disease. The prevalence of NASH in the general population is yet incompletely understood. The prevalence of nonalcoholic fatty liver disease (NAFLD) is better understood which can be detected noninvasively. The major risk factors for NAFLD are central obesity, type 2 diabetes mellitus, dyslipidemia, and metabolic syndrome. NAFLD is the most common liver disorder in western industrialized countries, affecting 20 to 40 percent of the general population [13]. Estimates of current prevalence range from 5 to 30 percent in the Asia-Pacific region, depending on the population studied [14, 15]. In our population, data is lacking. This study will help us to help finding not only the data in adult population. However, NAFLD is probably the most common cause of liver disease in the preadolescent and adolescent age groups, but the majority of cases occur between the ages of 40 and 60. NAFLD and NASH are more common in men. The male predilection may reflect that men are more likely to fulfill criteria for the metabolic syndrome which will also help us find the strategies for the management of such problems. 

 Most patients with NASH are asymptomatic although fatigue, malaise, and vague right upper abdominal discomfort bring some patients to medical attention [1]. The most common presentation is elevation of liver aminotransferases detected on routine laboratory testing. Hepatomegaly is a frequent finding. Serum AST and ALT are elevated in almost 90 percent of patients [1]. The AST/ALT ratio is usually less than 1; this is much lower than the ratio in alcoholic hepatitis, which is usually above 2 and averaged 2.85 in one report and 2.6 in another [16, 17].

 Various radiological methods can detect the presence of fat in the liver, but no imaging modality is able to differentiate between the histological subtypes of relatively benign nonalcoholic hepatic steatosis or more aggressive NASH [18]. Ultrasonography often reveals a hyperechoic texture or a bright liver because of diffuse fatty infiltration [19]. However, this is a nonspecific finding and should not be used to make the diagnosis of NAFLD. Both CT, and MRI can identify steatosis but are not sufficiently sensitive to detect inflammation or fibrosis [20]. Magnetic resonance spectroscopy has the advantage over the other commonly used imaging modalities of ultrasonography, CT and MRI, as it is quantitative rather than qualitative or semiquantitative. One of the difficulties in determining test characteristics of the imaging tests for ruling in or ruling out NAFLD is that not all patients undergo confirmation by liver biopsy.

The indications for liver biopsy for suspected NAFLD have not been established in either children or adults and remain controversial. Liver biopsy is the only way to reliably distinguish between simple steatosis steatohepatitis, and fibrosis, and can also be helpful in excluding other causes of elevated serum aminotransferases. On the other hand, because there is currently no specific treatment for NAFLD other than weight management, the results of a liver biopsy are not likely to provide clinical benefit. Therefore, the benefits of performing a liver biopsy may not outweigh the costs and risks, unless there are specific features that suggest more severe or progressive liver disease.

 Although the etiology of NASH is unknown, it is frequently associated with obesity, type 2 diabetes mellitus, and hyperlipidemia. The constellation of abdominal obesity, hypertension, diabetes, and dyslipidemia has been called the metabolic syndrome, syndrome X, the deadly quartet, the insulin resistance syndrome, and the obesity dyslipidemia syndrome. One study used this definition to seek the metabolic syndrome in 304 consecutive patients with NAFLD of whom 120 (74 percent) were diagnosed with NASH. Metabolic syndrome was significantly more common in patients with NASH compared with those with fatty liver alone (88 versus 53 percent). Obesity has been reported in 69 to 100 percent of cases. Type 2 diabetes, a frequent complication of obesity, has been described in 34 to 75 percent of patients with NASH. Hyperlipidemia (hypertriglyceridemia and/or hypercholesterolemia), which is frequently associated with both obesity and type 2 diabetes, has been reported in 20 to 80 percent of patients with NASH.

 There is no proven effective therapy for NASH although the modification of risk factors, such as obesity, hyperlipidemia, and poor diabetic control, is generally recommended. Weight loss is the only therapy with reasonable evidence supporting benefit. Weight management is the only established treatment for NAFLD; small nonrandomized studies in children have shown improvement in liver histology or aminotransferase activity after weight loss [21–23]. Emphasis on physical activity seems appropriate because of its utility in improving insulin sensitivity, which appears to be an important pathway for the development of NAFLD. 

## 5. Recommendations

Nonalcoholic fatty liver disease is unexpectedly a common clinical problem which otherwise comes in to light when an individual undergoes investigations for any medical checkup.

 It is commonly associated with other metabolic illnesses like diabetes mellitus, obesity, hyperlipidemias, and so forth. 

It can lead to fibrosis cirrhosis and hepatocellular carcinoma.

There is little or no role of drugs which can modify the course of the disease. However, only the diet modifications and exercise have a definitive role in the treatment of the potentially dangerous condition.

## Figures and Tables

**Table 1:** Some important clinical features.

	No. of individuals (*n* = 135)
NAFLD	129
Hepatitis B&C	06
Fatty change on usg	86
BMI (Mean)	27.1
ALT bet 50–100 u/L	113
ALT bet 100–150 u/L	16
Mean Age yrs	39.2
